# The Role of BRCA Status on the Prognosis of Patients with Epithelial Ovarian Cancer: A Systematic Review of the Literature with a Meta-Analysis

**DOI:** 10.1371/journal.pone.0095285

**Published:** 2014-05-01

**Authors:** Chaoyang Sun, Na Li, Dong Ding, Danhui Weng, Li Meng, Gang Chen, Ding Ma

**Affiliations:** Department of Obstetrics and Gynaecology, Tongji Hospital, Tongji Medical College, Huazhong University of Science and Technology, Wuhan, Hubei, China; University of Texas Health Science Center at San Antonio, United States of America

## Abstract

**Objective:**

The role of BRCA dysfunction on the prognosis of patients with epithelial ovarian cancer (EOCs) remains controversial. This systematic review tried to assess the role of BRCA dysfunction, including BRCA1/2 germline, somatic mutations, low BRCA1 protein/mRNA expression or BRCA1 promoter methylation, as prognostic factor in EOCs.

**Methods:**

Studies were selected for analysis if they provided an independent assessment of BRCA status and prognosis in EOC. To make it possible to aggregate survival results of the published studies, their methodology was assessed using a modified quality scale.

**Results:**

Of 35 evaluable studies, 23 identified BRCA dysfucntion status as a favourable prognostic factor. No significant differences were detected in the global score of quality assessment. The aggregated hazard ratio (HR) of overall survival (OS) of 34 evaluable studies suggested that BRCA dysfunction status had a favourable impact on OS (HR = 0.69, 95% CI 0.61–0.79), and when these studies were categorised into BRCA1/2 mutation and low protein/mRNA expression of BRCA1 subgroups, all of them demonstrated positive results (HR = 0.67, 95% CI: 0.57–0.78; HR = 0.62, 95% CI: 0.51–0.75; and HR = 0.51, 95% CI: 0.33–0.78, respectively), except for the subgroup of BRCA1 promoter methylation (HR = 1.59, 95% CI: 0.72–3.50). The meta-analysis of progression-free survival (PFS), which included 18 evaluable studies, demonstrated that BRCA dysfunction status was associated with a longer PFS in EOC (HR = 0.69, 95% CI: 0.63–0.76).

**Conclusions:**

Patients with BRCA dysfunction status tend to have a better outcome, but further prospective clinical studies comparing the different BRCA statuses in EOC is urgently needed to specifically define the most effective treatment for the separate patient groups.

## Introduction

Epithelial ovarian carcinoma (EOC) is the fifth leading cause of cancer death in women [1], and the five-year relative survival rates for the late stage of EOC were less than 10% between 2004 and 2008 [2]. Despite advances in surgery and the wide use of platinum-based chemotherapy, the long-term outcome remains poor as a result of recurrences and the emergence of drug resistance, necessitating the discovery of biomarkers for predicting which patients will benefit or not benefit from systemic chemotherapy. Moreover, the lack of active therapeutic agents for patients with platinum-resistant cancers impels researchers to discover novel molecular targets helping define subsets of patients who may benefit the most from specific treatment.

In 1996, a detailed case-control analysis reported that BRCA1/2 germline mutations were beneficial prognostic factors for patients with EOC [3]. Since then, many scientists have tried to discover the real association between BRCA1/2 germline mutation status and the prognosis of EOC in subsequent studies, generating conflicting results [3]–[8]. Although, the mechanism underlying the association between BRCA1/2 germline mutations and survival is not fully understood, i*n vitro* experiments have shown that BRCA1/2 deficient cells display a deficiency in repairing double-strand DNA breaks by homologous recombination [9]–[11]. This biological mechanism may be responsible for increased chemo-sensitivity, which results in a longer progression-free survival (PFS) and overall survival (OS) [12]. More inspiringly, BRCA1/2 mutation carries can obtain an excellent response from targeted therapies, such as the poly (ADP) ribose polymerase (PARP) inhibitor (Olaparib) [13], [14]. However, BRCA1/2 germline mutation carriers only account for 10% to 15% of EOCs. Fortunately, recent data suggest that many sporadic EOCs display “BRCAness”, or dysfunction of BRCA1/2. Additionally, in sporadic EOCs, low BRCA1 expression detected by immunohistochemistry (IHC) or RT-PCR or BRCA1 promoter methylation had also been reported as a clinically useful tool to provide important information on prognosis [15].

The aim of this study was to assess the role of BRCA dysfunction status, including BRCA1/2 germline/somatic mutations, low BRCA1 protein/mRNA expression or BRCA1 promoter methylation in sporadic EOCs, on prognosis in EOCs by carrying out a systematic review of the literature followed by a meta-analysis, and to estimate to what extent do these BRCA statuses influence patients’ prognosis.

## Methods

### Publication Selection

This study has been registered at the International Prospective Register of Systematic Reviews (PROSPERO, http://www.crd.york.ac.uk/prospero/display_record.asp?ID=CRD42011001747) in 2011. An electronic search of Medline, Embase, and CNKI (China National Knowledge Infrastructure) was used to select articles with the following keywords: ‘ovarian neoplasm’, ‘ovarian tumour’, ‘ovarian carcinoma’, ‘ovarian malignance’ or ‘ovarian cancer’ and ‘BRCA1’, ‘BRCA2’ or ‘BRCA*’ and ‘prognos*’, ‘surviv*’, ‘outcome’ or ‘marker’. This search strategy was complemented by examining the personal bibliography of the authors. To avoid overlap between patient populations, when authors reported results obtained on the same patient cohorts in several publications, only the most recent report or the most complete one was included in the analysis. The search was updated in September 2013. A study must have been published as a full paper in the English or Chinese language. To be eligible for inclusion, studies had to meet the following criteria: addressed epithelial ovarian cancer and analysed patients’ prognosis according to BRCA statuses (assessed BRCA1/2 mutations, assessed BRCA1/2 protein expression through IHC or assessed mRNA level through RT-PCR, and/or assessed BRCA1 promoter methylation in the primary tumour (not in metastatic tissue or in tissue adjacent to the tumour)). The primary outcome was overall survival (OS) and the secondary outcome was progression-free survival (PFS).

### Data Extraction and Methodological Assessment

The data retrieved from the reports included authors, years of studies and publications, patients’ resources, population size, methods, histology, stage and treatment. To avoid bias in the data abstraction process, three reviewers (Chaoyang Sun, Na Li, Dong Ding) abstracted the data independently and subsequently compared the results. All of the data were checked for internal consistency, and disagreements were resolved by discussion.

To assess methodology, three investigators (Chaoyang Sun, Na Li, Dong Ding) read each publication independently and scored them according to the European Lung Cancer Working Party (ELCWP) scoring scale, with some modification (Method S1 in File S1) [16]. The scores were compared, and a consensus value for each item was reached in meetings attended by at least two investigators. The score evaluates a number of aspects of methodology, which were grouped into four main categories: design, laboratory methods, generalisability of results and the analysis of the study data. Each category had a maximum score of 10 points, giving a theoretical total maximum score of 40 points. The final scores were expressed as percentages ranging from 0 to 100%, with higher values reflecting better methodological quality.

### Statistical Methods

A study was considered to be significant if the P-value for the statistical test comparing the survival distributions between the groups of BRCA dysfunction and normal BRCA status was <0.05. The study was called ‘positive’ when BRCA dysfunction status was found as a favourable prognostic factor for survival. Other situations were called ‘negative’, including when a significant survival difference was found, but the group of patients with BRCA dysfunction status fared worse.

Non-parametric tests were used to compare the distribution of the quality scores according to the value of a discrete variable (Mann-Whitney tests).

For the quantitative aggregation of the survival results, we measured the impact of BRCA dysfunction status on prognosis by the hazard ratio (HR) between the survival distributions of the two BRCA status groups. For each study, the HR was extracted or estimated by a method that depended on the results provided in the publication. The most accurate method was to retrieve the HR estimate and its variance from the reported results or to calculate it directly using parameters provided by the authors for univariate analysis: the confidence interval (CI) for the HR, the log-rank statistic, its *P*-value or the O-E statistic (difference between numbers of observed and expected events). If these parameters were not available, we evaluated the total number of events, the number of patients at risk in each group and the log-rank statistic or it’s *P*-value, allowing for the calculation of an approximation of the HR estimate. Finally, if the only useful data were in the form of graphical representations of the survival distributions, we extracted survival rates at specified times to reconstruct the HR estimate and its variance, with the assumption that during the study follow-up, the rate of patients censored was constant [17]. If this latter method was used, three independent persons read the curves to reduce imprecision in the reading variations.

If survival was reported separately for particular subgroups, these results were included in the meta-analysis of the corresponding subgroups. The same patients were never considered more than once in each analysis. The individual HR estimates were combined into an overall HR using the method published by Yusuf S and Peto R et al [18]. If the assumption of homogeneity had to be rejected, we used a random-effects model as a second step. By convention, an observed HR <1 implied better survival for the group with BRCA dysfunction status. This impact of BRCA status on survival was considered to be statistically significant if the 95% CI for the overall HR did not include 1.

Horizontal lines indicate the 95% CI, and each box represents the HR point estimate; the box size is proportional to the number of patients included in the study. A funnel plot and Begg’s linear regression test were used to investigate any possible publication bias [19].

For all analyses, a two-sided *P* value of <0.05 was considered to be statistically significant. All analyses were performed using SPSS version 13.0 (SPSS, Chicago, IL) and STATA version 10.0 software (Stata Corporation, College Station, TX).

Studies that were eligible for the systematic review were called ‘eligible’, and those providing data for the meta-analysis were called ‘evaluable’.

## Results

### Study Selection and Characteristics

**Figure 1:**
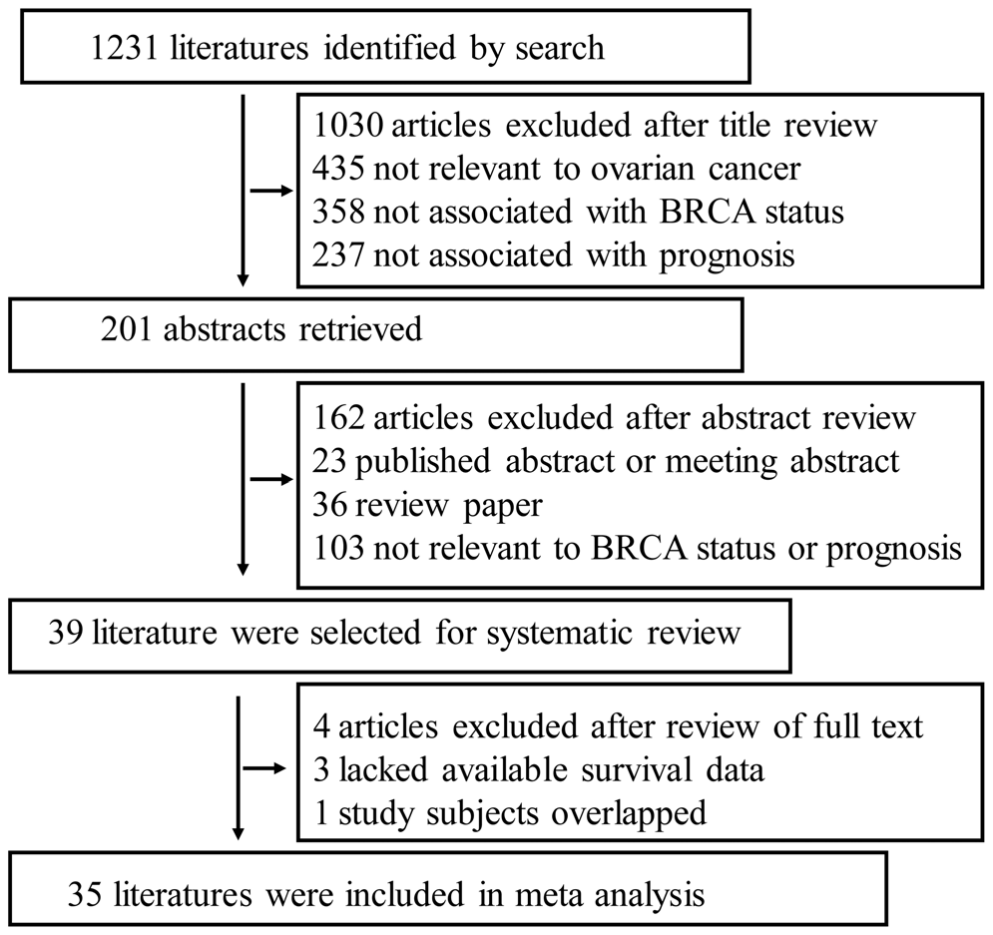
Flow chart of publication selection.

The primary search yielded a total of 1,231 publications, 1030 of which were excluded by title screening. Abstracts of the remaining 201 papers were reviewed, resulting in 162 being excluded and leaving 39 as candidate articles [3]–[8], [15], [20]–[51]. To reach a final decision on which articles were to be included in the meta-analysis, we examined all 39 papers in detail, which resulted in the further exclusion of 4 papers because survival information was not available for three papers [29], [35], [46] and one study’s [42] subjects overlapped with a subsequent study that the authors published six years later [26] (Figure 1). All eligible articles were reviewed by three independent investigators. The main features of the 39 studies eligible for the systematic review, which were published between 1996 and 2013, are shown in Table S1 in File S1. All of the eligible literatures were case-control studies. A total of 26 studies investigated BRCA1/2 germline and/or somatic mutions, while low BRCA1 protein/mRNA expressions and BRCA1 promoter methylation statuses in sporadic EOCs were studied in nine, two, and two studies, respectively.

As shown in Table 1, 26 studies were performed on BRCA1/2 germline and/or somatic mutions. Twenty-one (21/26, 80.8%) studies investigated the germline BRCA1/2 mutation alone, four (4/26, 15.4%) studies investigated BRCA mutation status including germline and somatic BRCA1/2 mutation together, and the one (1/26, 3.8%) study left investigated BRCA1 dysfunction secondary to germline, somatic BRCA1 mutation or BRCA1 promoter methylation. The detailed information of these 26 studies was listed in Table 1. Eighteen (18/26, 69.2%) papers identified BRCA1/2 mutation as a good prognostic factor for survival, while the remaining eight (8/26, 30.8%) concluded that BRCA1/2 mutation was not a prognostic factor for survival.

**Table 1:** Characteristics of studies of patients with BRCA1/2 mutated ovarian cancer.

First author	Study year,published year	Country	Histology	Stage	No. of cases/controls	Laboratorymethods	BRCA status	Germline/somatic	Mutation Types	Treatment	Survivalresult
Aida	1984–1996,1998	Japan	se	I–III	13/29	SCCP,PCR,seq	BRCA1	Germ	Deleterious	2	positive
Alsop	2002–2006,2012	Austrilia	se	I–IV	118/536	PCR,seq, MLPA	BRCA1/2	Germ	Deleterious	2	positive
Artioli	,2010	Italian	all	I–IV	48/40	PCR,seq	BRCA1/2	Germ	Deleterious	1+2	positive
Boyd	1986–1998,2000	U.S.A	all	I–IV	88/100	PCR,seq	BRCA1/2	Germ	Deleterious	2	positive
Brozek	1995–2004,2008	Poland	se+CCC	I–IV	21/130	DHPLC,RFLP,seq	BRCA1/2	Germ	Deleterious+ VUS	2	positive
Buller	1990–2000,2002	U.S.A	all	I–IV	59/59	PTT,SSCP,seq	BRCA1	Mixed	Deleterious+ VUS	2	negative
Cass	1990–2001,2003	U.S.A	all	III–IV	29/25	SSCR,seq	BRCA1/2	Germ	Deleterious	2	positive
Chetrit	1994–1999,2008	Israel	all	I–IV	213/392	PCR,seq	BRCA1/2	Germ	Deleterious	2	positive
David	1994–1999,2002	Israel	all	I–IV	229/549	SCCP,seq	BRCA1/2	Germ+Somatic	Deleterious	2	positive
Dann	1999–2007,2012	U.S.A	all	II–IV	15/38	PCR,seq	BRCA1/2	Germ+Somatic	Deleterious	2	negative
Gallagher	1996–2006,2011	U.S.A	all	III–IV	36/74	PCR,seq	BRCA1/2	Germ	Deleterious	2	positive
Hennessy	1990–2006.2010	U.S.A	all	I–IV	43/192	PCR,seq	BRCA1/2	Germ+Somatic	Deleterious	2	positive
Hyman	2001–2010,2012	U.S.A	se	III–IV	69/298	PCR,seq	BRCA1/2	Germ	Deleterious	2	positive
Johannsson	1958–1995,1998	Swedish	all	I–IV	38/97	PTT,SSCP,seq	BRCA1	Germ	Deleterious	2+3	negative
Lacour	1996–2007,2011	U.S.A	all	III–IV	95/183	PCR,seq	BRCA1/2	Germ	Deleterious	–	positive
Majdak	1997–2002,2005	Poland	se+Mu	I–IV	18/171	F-CSGE,seq	BRCA1	Germ	Deleterious	2	positive
McLaughlin	1995–1995,2002–2004,2012	Canada	all	I–IV	218/1408	PTT, seq,DGGE, DHPLC	BRCA1/2	Germ	Deleterious	2	negative
Pal	2000–2003,2007	West central Florida	all	III–IV	32/177	PCR,seq	BRCA1/2	Germ	Deleterious+ VUS	–	negative
Pharoah	,1999	U.K	all	I–IV	151/119	PTT,SSCP	BRCA1/2	Germ	Deleterious	–	negative
Ramus	1992–1997,2001	Israel	all	I–IV	27/71	SSCP,PCR,seq	BRCA1/2	Germ	Deleterious	–	negative
Rubin	,1996	U.S.A	all	III–IV	43/43	SSCP,PCR,seq	BRCA1	Germ	Deleterious+ VUS	–	positive
Tan	1991–2006,2008	U.K	all	III–IV	22/44	SCCP,seq	BRCA1/2	Germ	Deleterious+ VUS	2	positive
Vencken	1980–2009,2011	Netherlands	all	I–IV	112/222	PCR,seq	BRCA1/2	Germ	unknown	2	positive
Yang	,2011	multi-country	all	III–IV	59/251	exom seq	BRCA1/2	Germ+Somatic	Deleterious+ VUS	2	positive
Zweemer	,1999	Netherlands	–	–	42/84	PTT,PCR	BRCA1/2	Germ	Deleterious	–	positive
Zweemer	,2001	Netherlands	All	I–IV	23/17	PTT,PCR,seq	BRCA1/2	Germ	Deleterious	–	negative

**Histology**: pathological histology of ovarian cancer, se = serous ovarian cancer, CCC = clear cell cancer of, Mu = mucinous ovarian cancer. all = almost all of the epithelial ovarian cancer types, including serous, mucinous, clear cell cancer, etc.

**Laboratory methods**: laboratory methods used to detect BRCA1/2 mutation, PTT = Protein truncation test, SSCP = Single-Strand Conformation Polymorphism, seq = sequencing, DGGE = fluorescent multiplex denaturing gradient gel electrophoresis, MLPA = multiplex ligation-dependent probe amplification, DHPLC = Denaturing high performance liquid chromatography, RFLP = Restriction fragment length polymorphisms, F-CSGE = Fluorescence-based Conformation Sensitive Gel Electrophoresis.

**Germline/somatic**: Germ = germline mutation, Mixed = BRCA1 germline/somatic mutation or BRCA1 promoter methylation.

**Mutation types:** VUS = variants of unknown significance.

**Treatment**: chemotherapy used, 1 = only Platinum was used, 2 = Platinum-based chemotherapy, 3 = other agents without Platinum, like Paclitaxel, etc.

Immunohistochemistry (IHC) was used in 9 studies to detect the low expression of BRCA1 protein in sporadic EOCs. The MS110 clone antibody was used in 88.9% (8/9) of the studies. Various experimental procedures were performed with the same cut-off value (<10% positive cells) except for one study [49], and the summary proportion of low expression of BRCA1 (with cut-off value as <10% positive cells) in sporadic ovarian cancer was 55.2% (Table 2).

**Table 2:** Characteristics of studies of patients of ovarian cancer with low BRCA1 expression measured by IHC.

Firstauthor	Study year,published year	Country	stage	Histology	No. of cases/controls	Clone	dilution	Retrieval	readers	Doubleblind	Cutoff	Low%	treatment	Survivalresult
Weberpals	2008,2011	Canada	II–IV	all	75/41	MS110	1∶100	–	2	Yes	10%	65%	1	positive
Carser	1998–2004,2011	U.K	I–IV	all	120/172	MS110	–	steam heat	2	Yes	10%	41%	1	positive
Gan	1991–2007,2013	U.K	I–IV	se	112/19	MS110	1∶80	microwave	1	Yes	≤70*	84.4%	2	positive
Kaern	1990–1992,2005	Norway	III	all	30/16	MS110	1∶200	microwave		No	10%	65.20%	2	negative
Sirisabya	1996–1999,2007	Thailand	I–III	all	87/12	–	–	microwave	1	No	10%	87.80%	1	negative
Thrall	,2006	U.S.A	I–III	all	97/55	MS110	1∶50	steam heat	2	Yes	10%	63.8%	1	positive
Swisher	,2009	U.S.A	I–IV	all	39/76	MS110	1∶250	steam heat		No	10%	34%	2	positive
Radosa	2000–2005,2011	Germany	III–IV	all	12/15	MS110	1∶200	heat	2	Yes	10%	44.40%	1	positive
Yu	1996–1998,2005	China	I–IV	all	35/15	MS110	1∶100	–		–	10%	70%	2	negative

**Histology**: pathological histology of ovarian cancer, all = almost all of the epithelial ovarian cancer types, including serous, mucinous, clear cell cancer, etc, se = serous ovarian cancer.

**Treatment**: chemotherapy used, 1 = only Platinum was used, 2 = Platinum-based chemotherapy.

*BRCA1 (H-score≤70) defined as the BRCA1-deficient group.

BRCA1 mRNA expression and BRCA1 promoter methylation in sporadic EOCs were studied in two papers each. Both articles identified the low expression of BRCA1 mRNA as a significantly better predictor of prognosis, while the other two papers on BRCA1 promoter methylation showed negative results (Table 3).

**Table 3:** Characteristics of studies of patients of ovarian cancer with low BRCA1 mRNA expression or BRCA1 promoter methylation.

	First author	Study year, published year	Country	Histology	stage	No. of cases/controls	Methods of methylation detecting	Treatment	significance
RT-PCR	Quinn	,2007	U.K	all	I–IV	47/23		2	positive
	Weberpals	1997–2005,2009	Canada	all	II–IV	25/26		2	positive
methylation	Wiley	1991–2000,2006	Italy	all	I–IV	44/171	MSP	2	negative
	Chiang	1986–2001,2006	USA	–	I–IV	10/25	MSRE+ Southern blot+ MSP	2	negative

**Histology**: pathological histology of ovarian cancer, all = almost all of the epithelial ovarian cancer types, including serous, mucinous, clear cell cancer, etc.

**Methods of methylation detecting**: methods of methylation detecting, MSP = methylation-specific polymerase chain reaction (PCR) analysis, MSRE = methylation-sensitive restriction enzyme digestion.

**Treatment**: chemotherapy used, 2 = Platinum-based chemotherapy.

### Quality Assessment

Overall, the global quality assessment score, expressed as a percentage, ranged between 36.7% and 89.4%, with a median of 70.6% (Table S2A in File S1, mean ± SD values are shown).

No statistically significant difference of scores were found between the 35 evaluable and 4 non-evaluable studies. There was also no statistically significant difference between the scores of the 26 positive studies and 13 negative studies, except the positive ones had better sub-scores for laboratory methodology (*P* = 0.016). The difference in the global and four subgroup scores between the studies classified according to the types of BRCA dysfunction statuses was not significant.

Table S2B in File S1 shows the scores for the 35 studies classified as evaluable for the meta-analysis. There was no significant difference between significant and not significant studies in the global score, except for the sub-score of generalisability (*P* = 0.013). Moreover, the different types of BRCA dysfunction status did not affect the overall quality assessment or the four subgroup scores.

### Meta-analysis of BRCA Status and OS of Ovarian Cancer

The absence of significant qualitative differences between positive and negative trials allowed us to perform a quantitative aggregation of the survival data. Subgroup analysis was performed because the heterogeneity of the trials was obvious: the studies had reported on patients with different BRCA dysfunction statuses (BRCA1/2 germline/somatic mutations, low BRCA1 expression tested by IHC or RT-PCR, and BRCA1 promoter methylation in sporadic EOCs). In this study, we combined studies of germline, somatic BRCA1/2 mutations together as one intervention called the BRCA1/2 mutations in subgroup meta-analysis.

**Figure 2:**
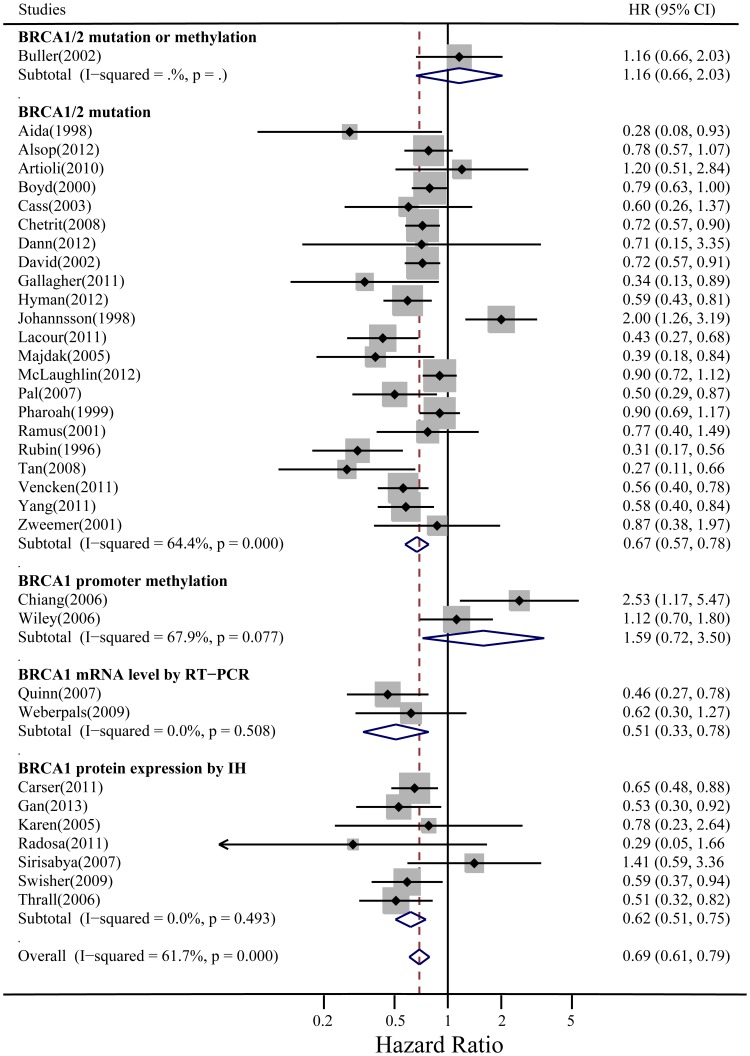
Summary hazard ratios (HRs) and 95% confidence intervals (CIs) of epithelial ovarian cancer OS for BRCA dysfunction status. Horizontal lines represent 95% CIs; diamonds represent summary estimates with corresponding 95% CIs. Test for heterogeneity: *P* = .000, *I* ^2^ = 61.7%. A random-effects model was used for analysis.

The overall meta-analysis of OS included 34 aggregable studies with 7,986 patients (one studies only provided PFS). The test of overall heterogeneity was significant (*I^2^* = 61.7%, *P* = 0.000), which primarily came from the BRCA1/2 mutation subgroup (*I^2^* = 64.4%, *P* = 0.000), while the heterogeneity of the remaining three subgroups (low BRCA1 expression by IHC or RT-PCR and BRCA1 promoter methylation in sporadic EOCs) was not significant. BRCA dysfunction status was associated with a better OS, with HR = 0.69, 95% CI: 0.61–0.79 in random-effects model (HR = 0.72, 95% CI: 0.61–0.79 in fixed-effects model). In the subgroup analyses according to different BRCA statuses, BRCA1/2 mutations (1,686 cases and 4,941 controls) and low BRCA1 expression by IHC (500 cases and 362 controls) or RT-PCR (72 cases and 49 controls) were statistically significantly better prognostic factors for survival (HR = 0.69, 95% CI: 0.59–0.80; HR = 0.62, 95% CI: 0.51–0.75; and HR = 0.51, 95% CI: 0.33–0.78 in the random-effects model, respectively; and HR = 0.72, 95% CI: 0.67–0.78; HR = 0.62, 95% CI: 0.51–0.75; and HR = 0.51, 95% CI: 0.33–0.78 in the fixed-effects model, respectively). However, BRCA1 promoter methylation (62 cases and 196 controls) was not associated with better prognosis (HR = 1.59, 95% CI: 0.72–3.50 in the random-effects model and HR = 1.40, 95% CI: 0.94–2.09 in the fixed-effects model) (Figure 2).

**Figure 3:**
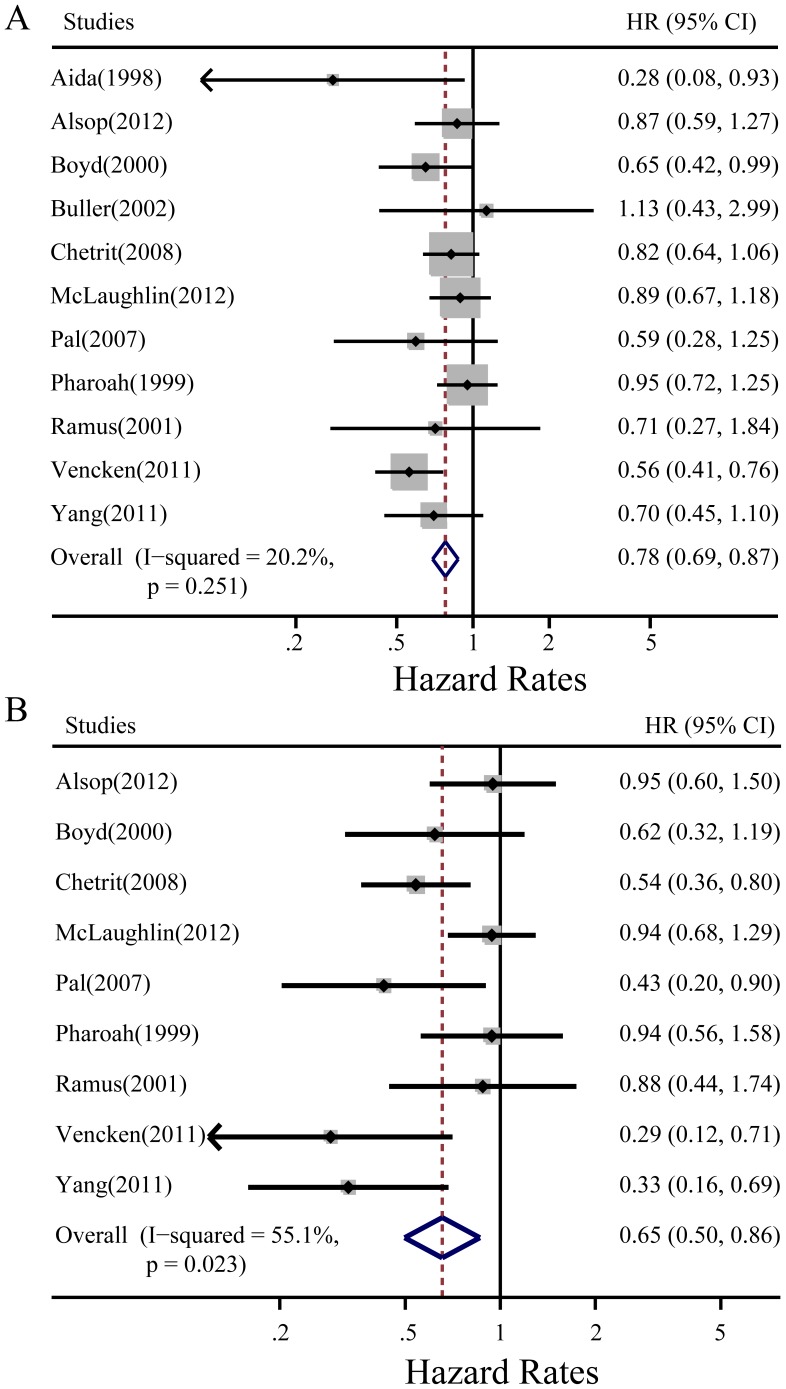
Subgroup meta-analysis of summary hazard ratios (HRs) and 95% confidence intervals (CIs) of ovarian cancer OS for different BRCA mutation statuses. **A:** BRCA1 mutation. **B:** BRCA2 mutation. Horizontal lines represent 95% CIs; diamonds represent summary estimates with corresponding 95% CIs. Test for heterogeneity: **A:** *P* = .251, *I* ^2^ = 20.2%, a fixed-effects model was used; **B:** *P* = .023, *I* ^2^ = 55.1%, a random-effects model was used.

When BRCA mutation was subdivided into BRCA1 or BRCA2 subgroups, the meta-analysis showed that both BRCA1 and BRCA2 mutations predicted better OS (HR = 0.78, 95% CI: 0.69–0.87 and HR = 0.65, 95% CI: 0.50–0.86 in a fixed and random-effects model, respectively) (Figure 3A, 3B).

### Meta-analysis of BRCA Status and PFS of Ovarian Cancer

**Figure 4:**
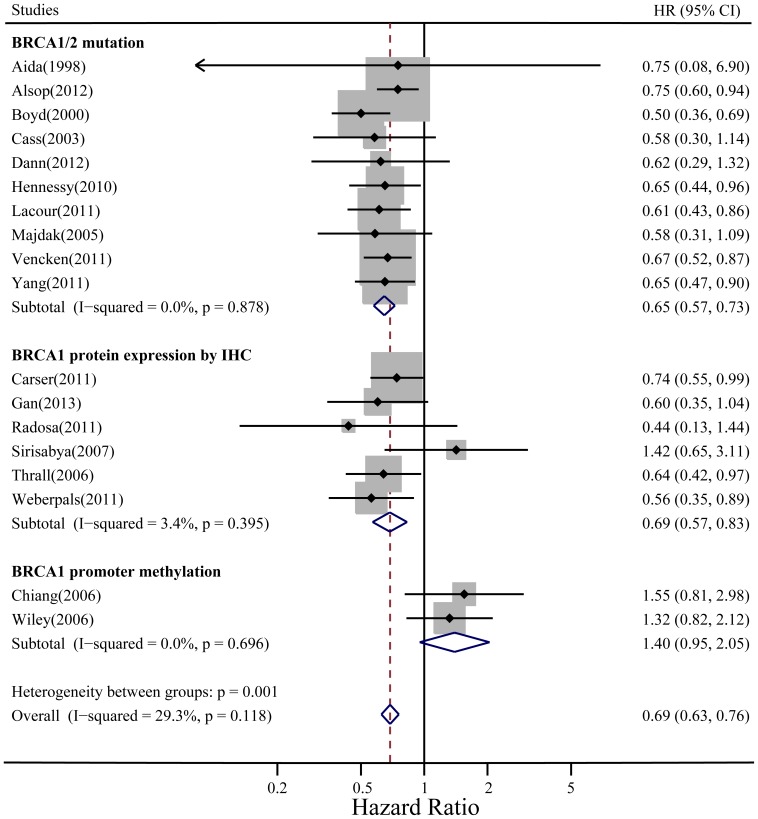
Summary hazard ratios (HRs) and 95% confidence intervals (CIs) of ovarian cancer PFS for BRCA dysfunction status. Horizontal lines represent 95% CIs; diamonds represent summary estimates with corresponding 95% CIs. Test for heterogeneity: *P* = .118, *I* ^2^ = 29.3%. A fixed-effects model was used.

The overall meta-analysis of PFS included 18 evaluable studies with 3,394 patients. The overall heterogeneity and the heterogeneity of all subgroups were not significant. BRCA dysfunction status was associated with a better PFS in ovarian cancer, with HR = 0.69 (95% CI: 0.63–0.76, fixed-effect model). In the subgroup analyses according to different BRCA statuses, BRCA1/2 mutation and low BRCA1 expression by IHC were statistically significant predictors for longer PFS (HR = 0.65, 95% CI: 0.57–0.73 and HR = 0.69, 95% CI: 0.57–0.83 in a fixed-effect model, respectively). However, BRCA1 promoter methylation was not associated with better PFS (HR = 1.40, 95% CI: 0.95–2.05) (Figure 4).

### Publication Bias

**Figure 5:**
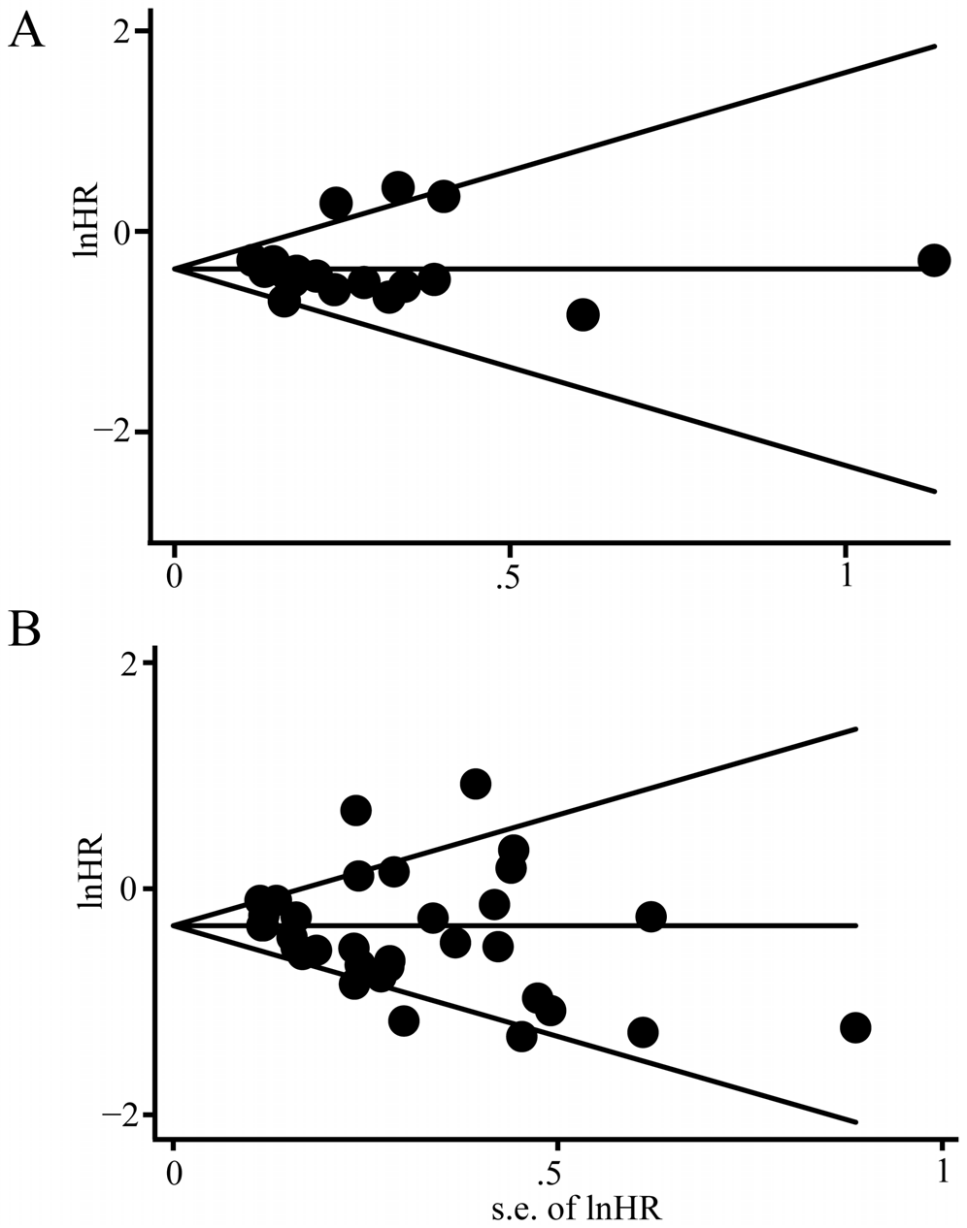
Begg’s funnel plots of the natural logarithm of the hazard ratios (HRs) and the SE of the natural logarithm of the HRs for all of the included studies reported with OS and PFS. A: Begg’s funnel plots for all of the included studies reported with OS, the dashed line represents 95% confidence intervals (CIs). Circles represent individual studies. Begg’s test: *P* = 0.221. B: Begg’s funnel plots for all of the included studies reported with PFS, the dashed line represents 95% confidence intervals (CIs). Circles represent individual studies. Begg’s test: *P* = 0.880.

Publication bias statistics were determined using Begg’s linear regression test. No publication bias was found for the studies used for the meta-analysis for overall survival (Begg’s test, *P* = 0.221) (Figure 5A); moreover, there is no publication bias was found for the studies used for the meta-analysis for PFS (Begg’s test, *P* = 0.880) (Figure 5B).

## Discussion

Our systematic review of the literature and meta-analysis demonstrate an improved prognosis in patients whose EOC display BRCA1/2 dysfunction, relative to those whose EOC display normal BRCA1/2 function. Although the comparison of prognostic benefit between BRCA1 and BRCA2 mutation was not feasible, the aggregated HRs indicated that patients with a BRCA2 mutation (HR = 0.65) may have a better prognosis than patients with a BRCA1 mutation (HR = 0.78). During the preparation of this manuscript, Bolton et al also reported BRCA1/2 germline mutation was associated with improved survival and BRCA2 carriers had the best prognosis, these findings are consistent with our results [52].

Although BRCA1/2 germline mutation carriers only account for small proportions of EOCs, fortunately, it has been estimated that approximately 50% sporadic EOCs show dysfunction of BRCA1/2 through different mechanisms. Our study is the first meta-analysis, to our knowledge, to assess if low BRCA1/2 expression status of sporadic EOCs could show similar effects on prognosis to BRCA1/2 mutation carriers. Our results showed that low BRCA1 expression measured by IHC or RT-PCR but not BRCA1 promoter methylation is a good prognostic factor for both OS and PFS in patients of sporadic EOCs, indicating that low BRCA1 expression status in sporadic EOCs show similar clinical effects on prognosis to germline mutations carriers. However, it is still difficult to draw a definite conclusion because these results were based on small numbers and require confirmation in larger studies, especially for low BRCA1 expression measured by RT-PCR and BRCA1 promoter methylation status. Swisher et al had stated that BRCA1 promoter methylation only occurs in a small proportion of sporadic ovarian cancer with low BRCA1 expression [33], therefore, other mechanisms that could cause low BRCA1 expression need to be further investigated.

In our study, patients whose EOC displays BRCA dysfunction had a favourable prognosis. BRCA1/2 gene products play a pivotal role in DNA repair mechanisms. The better prognosis of patients with BRCA dysfunction may be explained by their inability to repair double-strand DNA breaks caused by platinum-based chemotherapy. As we mentioned above, although the comparison of prognostic benefit between the BRCA1 and BRCA2 mutation was not feasible, the aggregated HR for OS for BRCA2 mutants was lower than that for BRCA1 mutants. It has been established by several research groups that BRCA2-mutated cells are recombination deficient and undergo significantly reduced homologous recombination repair of DNA double-strand breaks [13], [53]. Functionally, the primary function of BRCA2 appears to be regulation of the RAD51 protein, which is required for double-strand break repair by homologous recombination [10], indicating that BRCA2 lesions cause more substantial homologous recombination defects than BRCA1 lesions, because BRCA1 is more versatile. However, to date, there are no reports regarding the association between low BRCA2 expression and the prognosis of patients with sporadic EOCs. So, large population-based studies are urgently needed to discover the proportion of low BRCA2 expression patients among sporadic EOCs and the real role of low BRCA2 expression status on survival.

Our results may have important implications for the clinical management of EOCs. Ovarian carcinoma is clinically highly heterogeneous. Our study revealed that both BRCA1/2 mutations and low BRCA1 expression are associated with favourable survival in EOC, so, these BRCA statuses can guide choice of post-operative treatment decisions. Additionally, it has been demonstrated that a deficiency of the BRCA gene confers substantial sensitivity to a new class of agents, namely poly-ADP-ribose polymerase-1 (PARP1) inhibitors [13], [14]. A number of phase I and II studies have reported the successful applications of PARP inhibitors in BRCA1/2 mutation carries of ovarian and breast cancer, and phase III studies are underway [13], [14], [54]. So, routine testing BRCA1/2 germline mutation status of EOCs may now be warranted. A large proportion of sporadic EOCs demonstrate BRCA deficiency, whether these patients could also benefit from PARP1 inhibitor are still unclear. Moreover, a reliable assay to detect these patients is required. Our meta-analysis supports the IHC technique as a promising assay to detect a portion of sporadic ovarian cancer displaying BRCAness. Although RT-PCR may also be a potential assay to discover the BRCAness, the supporting positive literature was limited and without standard experimental protocols and uniform cut-off values. Large prospective clinical trials are expected for further validation.

In conclusion, EOCs patients with BRCA dysfunction status have better outcomes, but more fundamental studies and further prospective clinical studies are urgently needed. Furthermore, EOCs should be stratified by different BRCA statuses to specifically define the most effective treatment for the separate patient groups in further clinical studies.

## Supporting Information

File S1 Contains the following files: **Method S1:** The quality score for methodology modified according to the European Lung Cancer Working Party (ELCWP) scoring scale [^1^]. **Table S1:** Main characteristics and results of eligible studies. **Table S2:** Methodological assessment.
(DOC)

Checklist S1 PRISMA checklist.
(DOC)
