# Supplementary material for: The Role of BRCA Status on the Prognosis of Patients with Epithelial Ovarian Cancer: A Systematic Review of the Literature with a Meta-Analysis
Source: PLoS One. 2014 May 1;9(5):e95285. doi: 10.1371/journal.pone.0095285 (PMC4006804; doi:10.1371/journal.pone.0095285)
Supplement: File S1 — Contains the following files: Method S1: The quality score for methodology modified according to the European Lung Cancer Working Party (ELCWP) scoring scale [1]. Table S1: Main characteristics and results of eligible studies. Table S2: Methodological assessment. (DOC) [file pone.0095285.s001.doc]

**Methods S1: The** **quality score for methodology modified according to the European Lung Cancer Working Party (ELCWP) scoring scale^[1]^.**

Except when specified, the attributed value per item is 2 points if it is clearly defined in the article, 1 point if its description is incomplete or unclear and 0 point if it is not defined or is inadequate**.**

**Scientific design**

1. Study objective definition.
2. Study design: prospective (2 points); retrospective (1 point); not defined (0 point).
3. Outcome definition.
4. Statistical considerations: fully reported with a preliminary assessment of the patient/sample number to be included and/or analysed (2 points); patient/sample number to be included and/or analysed justified by the number of studied variables (minimum 10 patients per variable) (1 point); not defined (0 point).
5. Statistical methods and tests description.

**Laboratory methodology**

1. Blinding in the biological assays performance: double-blind (2 points); simple-blind (1 point); unblinded or not defined (0 point).
2. Tested factor description: DNA (types of exons analysed), messenger RNA (complete or partial with description of the primers used), protein (nuclear, cytoplasmic or extracted from cellular components), antibodies (type of tissue or liquid sampled).
3. Tissue sample conservation: either fresh tissue or conservation requiring freezing at ≤−80°C in presence of an anti-RNAase for RNA or freezing at ≤−20°C for DNA, protein and serum, or fixation in formol, alcohol or paraffin.
4. Description of the revelation test procedure of the biological factor: PCR with mention of primers, polymerase type, general reaction conditions (concentration of the various reagents, cycle number, duration and temperature of the various steps); DNA-;sequencing with the method used, the electrophoresis characteristics (gel composition, duration, temperature and generator voltage) and revelation procedure; reverse transcription (RT) with the transcriptase-reverse type and general conditions of incubation (reagent concentration, temperature and duration); SSCP with the gel composition (acrylamide percentage, glycerol content), other electrophoresis characteristics (duration, temperature and generator voltage), coloration method (the SSCP must be followed by the abnormally migrated fragments sequencing if there is no negative internal control); DGGE with gel composition and gel composition gradient, other electrophoresis characteristics (duration, temperature and generator voltage) and coloration method (the DGGE must be followed by the abnormally migrated fragments sequencing if there is no negative internal control); restricted fragment length polymorphism (RFLP) with the restriction enzyme type, temperature and incubation time, electrophoresis characteristics (gel composition, duration, temperature and generator voltage), coloration method (the RFLP must be followed by the abnormally migrated fragments sequencing if there is no negative internal control); IHC with the first antibody type and clone identification, second antibody type, reaction characteristics (antibodies concentration, duration and temperature of incubation), colouration method (peroxydase, alkaline phosphatase or chromagenic method), epitope unmasking method in case of fixed tissue, endogenous peroxydase activity inhibition method if the colouration method requires peroxydase; ELISA with the type of antibodies/antigens used, general reaction conditions (reagents concentration, temperature, duration), unspecific sites blockage (type of serum used), colouration and reading methods (fluorometry, spectrophotometry, *etc.*); Western blot with types of antibodies/antigens used, gel composition, other electrophoresis characteristics (duration, temperature, generator voltage), colouration method; immunoblot with the type of antibodies/antigens used, the type of membrane, other electrophoresis characteristics (duration, temperature, generator voltage), colouration method.
5. Description of the negative and positive control procedures.

**Generalizability**

1. Patient selection criteria, including histological type, disease stage and treatment.
2. Patients' characteristics, including histology type, disease stage and treatment.
3. Treatment description.
4. Source of samples.
5. Number of unassessable samples with exclusion causes.

**Results analysis**

1. Follow-up description, including the number of events.
2. Statistics description
3. Survival analysis according to the biological marker.
4. Univariate analysis of the prognostic factors for survival: report of the relative risk with the confidence interval (2 points); results without evaluation of the relative risk and its confidence interval (1 point); not reported or inadequate (0 point).
5. Multivariate analysis of the prognostic factors for survival: report of the relative risk with the confidence interval (2 points); results without evaluation of the relative risk and its confidence interval (1 point); not reported or inadequate (0 point)

**Reference**

**[1]** Steels E, Paesmans M, Berghmans T, Branle F, Lemaitre F, et al. (2001) Role of p53 as a prognostic factor for survival in lung cancer: a systematic review of the literature with a meta-analysis. Eur Respir J 18: 705-719.

**Table S1: Main characteristics and results of eligible studies**

|  | All study | | BRCA1/2 mutation | | BRCA1 IHC | | BRCA1 mRNA | | BRCA1 promoter methylation | |
| --- | --- | --- | --- | --- | --- | --- | --- | --- | --- | --- |
|  | Total | E | Total | E | Total | E | Total | E | Total | E |
| NO. | 39(26) | 35(23) | 26(18) | 23(15) | 9(6) | 8(6) | 2(2) | 2(2) | 2(0) | 2(0) |

E= number of studies evaluable for meta-analysis; ( ) = number of studies identifying BRCA status as a statistically significant good prognostic factor.

**Table S2: Methodological assessment**

|  | Global score (%)  Mean(SD) | Design (/10) | Laboratory  methodology (/10) | Generalisabilty  (/10) | Results analysis  (/10) |
| --- | --- | --- | --- | --- | --- |
| **A: All trials (n=39)** |  |  |  |  |  |
| Total | 67.3(14.3) | 7.5(1.0) | 7.5(1.7) | 6.2(2.1) | 5.7(2.6) |
| Evaluable for the MA(n=35) | 68.5(14.1) | 7.6(0.8) | 7.6(1.7) | 6.4(2.2) | 5.8(2.6) |
| Not evaluable for the MA (n=4) | 56.6(12.6) | 7.3(2.1) | 6.8(1.9) | 4.6(0.5) | 4.1(2.4) |
| P-value | 0.092 | 0.982 | 0.381 | 0.179 | 0.195 |
| Significant results(n=26) | 70.6(12.5) | 7.7(0.9) | 7.7(1.7) | 6.8(1.9) | 6.0(2.6) |
| Not significant results(n=13) | 60.8(15.9) | 7.3(1.0) | 7.0(1.8) | 5.0(2.2) | 5.0(2.7) |
| P-value | 0.294 | 0.187 | **0.016** | 0.231 | 0.058 |
| BRCA mutation(n=26) | 67.3(15.2) | 7.6(1.1) | 7.4(1.7) | 6.2(2.2) | 5.7(2.7) |
| Others (n=13) | 67.4(13.0) | 7.5(0.8) | 7.8(1.9) | 6.2(2.0) | 5.5(2.7) |
| P-value | 0.735 | 0.435 | 0.918 | 0.872 | 0.988 |
| **B: Evaluable trails (n=35)** |  |  |  |  |  |
| Total | 68.5(14.2) | 7.6(0.8) | 7.6(1.7) | 6.4(2.2) | 5.8(2.6) |
| Significant results(n=23) | 71.9(12.4) | 7.7(0.7) | 7.8(1.8) | 7.1(1.8) | 6.2(2.6) |
| Not significant results(n=12) | 62.2(15.7) | 7.4(1.0) | 7.3(1.6) | 5.0(2.3) | 5.2(2.7) |
| P-value | 0.068 | 0.503 | 0.294 | **0.013** | 0.263 |
| BRCA mutation(n=23) | 68.1(15.7) | 7.6(0.9) | 7.3(1.8) | 6.4(2.3) | 5.9(2.7) |
| Not BRCA mutation(n=12) | 69.4(11.3) | 7.6(0.7) | 8.1(1.6) | 6.3(2.1) | 5.7(2.6) |
| P-value | 0.878 | 0.878 | 0.248 | 0.905 | 0.959 |
|  |  |  |  |  |  |

(A) all trials and (B) evaluable trials for meta-analysis
